# Experiences, perceptions and unexpressed needs of patients undergoing heart and lung transplantation in intensive care unit: a qualitative phenomenological study

**DOI:** 10.3389/fpsyg.2025.1646086

**Published:** 2025-09-09

**Authors:** Nikita Valentina Ugenti, Silvio Quirini, Marianna Aleandri, Vittorio Di Filippo, Stefano Durante, Alice Ferretti, Carolina Guerrieri, Carmela Martella, Angela Peghetti, Silvia Sgarzi, Sara Valentini, Angela Vetromile, Elena Lia

**Affiliations:** ^1^IRCCS Azienda Ospedaliero-Universitaria di Bologna, Bologna, Italy; ^2^Ospedale Privato Accreditato Villa Laura, Bologna, Italy

**Keywords:** transplant, intensive care unit, patient experience, caring, patient-centered care

## Abstract

**Introduction:**

Patients in intensive care units require advanced clinical care as well as attention to psychological social and emotional needs, often overlooked. Heart and lung transplant recipients experience a particularly complex postoperative journey, marked by physical fragility, emotional vulnerability, and identity transformation. Communication barriers caused by sedation, intubation, and disorientation, combined with a focus on physiological stability, hinder understanding of their lived experience. A lack of qualitative research in this topic limits the development of person-centered care and mismatches between professional priorities and patient needs may lead to depersonalization and dissatisfaction. This study aimed to explore ICU experiences of transplant patients through the richness and complexity of their individual journey.

**Methods:**

A descriptive phenomenological study was conducted at IRCCS University Hospital of Bologna. Semi-structured interviews were performed with 21 heart (average ICU stay: 6 days) and lung (average ICU stay: 13 days) transplant recipients, 2–4 days post-ICU discharge. Interviews aimed to capture patients’ recollections while ensuring clinical stability. Thematic content analysis was used to identify key themes.

**Results:**

Six main themes emerged: (1) care environment, (2) sensory perceptions, (3) person’s empowerment, (4) lived experiences, (5) transplant path, and (6) quality of care. Patients reported feelings of isolation, disorientation, frustration and impaired communication due to sedation and intubation. Emotional experiences ranged from fear and loneliness to hope and gratitude. Reflections on the donor revealed ambivalent emotions including guilt and appreciation. Personalized care, empathetic communication, and supportive relationships with healthcare professionals were seen as essential for emotional well-being and recovery.

**Discussion:**

Heart and lung transplantation is a deeply transformative experience. Beyond clinical care, patients need emotional and psychological support. Personalized, empathetic interventions and improved communication strategies are crucial to enhancing both recovery outcomes and the overall ICU experience.

**Clinical trial registration:**

ClinicalTrials.gov, identifier NCT06773052.

## 1 Introduction

Subjects hospitalized in intensive care unit (ICU) represent a particular category of patients, as the high complexity of the assistance required by their condition is accompanied by equally important psycho-social needs, which are not always fully understood and satisfied by the healthcare team. The relevant causes are to be found in the difficult approach and communication methods toward subjects who are sometimes analgo-sedated, intubated, tracheostomized, mechanically ventilated (Karlsen et al., 2023); as well as in less attention given to their psychological-relational needs, because of the urgency to prioritize critical care (Thomas et al., 2017) and, finally, in the lack of knowledge and specific training of staff relating to the experience and perceptions of patients in ICU. Therefore, it is very likely that healthcare professionals ground treatments on their own perceived priorities, rather than on the patients and their needs, which sometimes leads to an unsatisfactory and depersonalized care (Berntzen et al., 2020; Thomas et al., 2017). Additionally, existing studies do not provide a global and consistent vision of the personal meaning of the experience and the unexpressed needs of users, especially with regard to transplant recipients (Sheikhalipour et al., 2018) or rather, in detail, heart and lung transplant recipients (Dixit et al., 2024; Stubber and Kirkman, 2020), which represent the population of interest of our study. Transplant recipients constitute a particularly delicate category of patients, as their hospitalization in ICU marks the first step toward recovery after a period in which they lived in fear, caused by the terminal stage of their underlying pathology (Sheikhalipour et al., 2018). Another peculiar aspect that characterizes this type of patient is the loneliness and deprivation of human contact with their family members, a condition experienced on a daily basis during the first phase subsequent to the transplant, as a result to the protective isolation for immunosuppression. Protective isolation is perceived both as a necessary shield against infection and as a source of emotional distress and profound loneliness (Biagioli et al., 2019; Biagioli et al., 2017). This is sometimes compared by patients to a prison (Thain and Gibbon, 1996) and can increase levels insomnia, anxiety and depression (Li et al., 2023). On the other hand, research into patients’ experiences, emotions and thoughts in the first 4 weeks after transplantation appears to be limited. Most studies are conducted several months or even years after the transplantation (Nåden and Bjørk, 2023), thus losing the focus on assistance in ICU. These reasons, combined with the clinical complexity of the type of user in question, lead to a particularly complex post-operative management, both from a technical and psychosocial point of view (McCurry, 2019). Been-Dahmen et al. (2018) found that transplant patients want to be given knowledge and instructions about their condition, share personal experiences with other patients, and discuss clinical, emotional and social issues with nurses Therefore, the use of generic or “one-size-fits-all” care, without personalization, is considered inadequate to meet their needs (Fatma et al., 2021; Been-Dahmen et al., 2018). Understanding and awareness of transplant patients’ experiences in ICU would allow staff to be adequately trained and to introduce new measures customized to the actual needs of users and provide quality care (Fatma et al., 2021; Stubber and Kirkman, 2020). Furthermore, it is necessary to take into consideration that the coherent and prompt response to the requests of hospitalized patients can decrease anxiety and recovery time (Joo et al., 2024). The incorrect identification of needs regarded as important amplifies subjective stressors and reduces long-term quality of life; consequently, individual care shall include those patient-identified preferences (Schindler et al., 2013). Therefore, a qualitative phenomenological study was conducted to investigate, through semi-structured interviews, the experience of patients undergoing heart and lung transplantation within the specific context of Cardio-thoracic and Vascular Anesthesia and Intensive Care Unit (CTV ICU) of the IRCCS University Hospital of Bologna. The study aims at helping nurses to provide more adequate care, to meet the individual needs of patients and describe the meaning and essence of the hospitalization experience, through the in-depth analysis of the specific words pronounced by the patients themselves.

## 2 Materials and methods

A total of 21 participants were included in the study, with sampling continuing until data saturation was reached.

### 2.1 Aim

The aim of this study was to investigate the experience, the perceptions and the unexpressed needs of patients undergoing heart and lung transplants who are admitted to the CTV ICU of IRCCS University Hospital of Bologna. The focus of this study was directed toward exploring the overall experience of patients who had undergone an intensive care stay, with particular attention to how they perceived their hospitalization and what emotional, relational, and psychological needs may have remained unmet during that period. The research aimed to understand not only the explicit narratives shared by the patients but also the underlying perceptions and subjective meanings they attributed to their time in the ICU.

### 2.2 Design

This study adopts an observational, prospective, and qualitative design, grounded in a descriptive phenomenological approach. Each patient enrolled in the study participated in a single, individual semi-structured interview, conducted in a private setting to facilitate open and reflective dialog.

### 2.3 Theoretical framework

The choice of the phenomenological method, as proposed by Burns and Grove (1997) and Rousseau and Saillant (1999), allowed for in-depth of the essence of the experiences that patients go through and the meanings they attribute to them (Del Barrio et al., 2004). The environment considered in the study contains subjects who are characterized by heterogeneity and diverse ways of experiencing life. The research group therefore decided to conduct a qualitative, phenomenological, descriptive observational study which, through semi-structured interviews, was able to investigate the experience of the patient undergoing heart and lung transplantation within the specific context of the CTV ICU with the aim of helping nurses to provide more adequate care and to meet the individual needs of the patient. Therefore, the study allowed for the formulation of a descriptive theory that can lead to a better understanding of the meaning of this human experience, discovering and describing its essence, through the in-depth analysis of the words of the patients themselves.

### 2.4 Study setting and recruitment

The study was conducted in the CTV ICU of IRCCS University Hospital of Bologna, one of the most important clinical centers in Italy for the treatment of heart and lung diseases. Participants were selected using purposive sampling, aimed at capturing a wide range of individual experiences related to the phenomenon under investigation. A total of 21 patients were recruited. Patient recruitment continued until data saturation was reached: when participants’ descriptions became repetitive, dominant themes were recurrent, and no new information emerged from subsequent interviews. Participant recruitment included all the patients who met the study inclusion criteria. The sample was intentionally heterogeneous, reflecting the natural diversity of the patient population in terms of medical condition, transplant type (heart or lung), age, gender, and ethnicity. This diversity allowed the study to explore the phenomenon from multiple perspectives, enriching the depth and relevance of the findings. Patient recruitment took place 1 to 2 days after discharge from the CTV ICU to the Cardiac Surgery Unit (CSU). Patients who met the inclusion criteria were approached by a member of the research team and provided with a detailed explanation of the study objectives, procedures, and ethical safeguards. Written informed consent was obtained from all participants. A total of 24 patients were excluded for various reasons: 4 declined to participate, 3 had linguistic barriers (e.g., non-italian speaking); 4 had tracheostomy, 2 had cognitive impairment, 6 died during their ICU stay, 1 withdrew from the study and 4 were not approached in time by the research team. Once informed consent was obtained, the interviews were conducted 2–4 days after the discharge from the CTV ICU, as indicated by the evidence available in the literature (Maartmann-Moe et al., 2021; Chang et al., 2012), in CSU. This time range was considered ideal both to allow patients to retain vivid memories of their experiences and to ensure that their clinical condition was stable.

### 2.5 Inclusion and exclusion criteria

**Inclusion criteria:**All patients undergoing heart or lung transplantation admitted to the CTV ICU of IRCCS University Hospital of Bologna were included in the study, with the following inclusion criteria:

- Aged ≥ 18 years.
- Able to understand and communicate in Italian.
- Transferred from ICU to CSU.
- Not affected by cognitive disorders.
- And who provided informed consent to the study.

**Exclusion criteria:** none.

### 2.6 Data collection

The interviews were planned so as not to burden nursing activities and not to interrupt patients’ meals or rest periods. The interviewers, trained in qualitative nursing research (N.V.U., C.M., E.L., S.Q., S.V.) had no prior relationship with any of the study participants. At the start of each interview, the interviewer introduced himself/herself by name and role. The length of the interviews was not decided *a priori* but by the participant. To respect patients’ needs, no minimum duration was set, however, the interviews lasted about an hour. Each interview was conducted once per patient and was not repeated. Privacy and confidentiality were ensured, as transplant patients are placed in single rooms to maintain protective isolation against infection. Participants were alone with the interviewer. Following the semi-structured interview technique, the researcher used a list of questions served to guide the interviews so they could remain centered on the topic. The aim was to investigate the phenomenon through open questions; the order of them was not structured, but followed the flow of the conversation, in order to guarantee high flexibility and adaptability. No pilot interviews were carried out prior to data collection. The data collection lasted 1 year.

Below are reported the guiding questions used as an outline for the interviews.

Questions related to the patient’s general experience in ICU and to the relationship with the healthcare team:

Tell me about your experience in the ICU. Why were you admitted to this unit? How long? What is the thing you remember most clearly? Are there any events or situations that you remember vaguely?

Have you experienced particularly positive or, on the contrary, particularly negative episodes or situations? What worked well for you? What would you have wanted to be different? What could be improved?

How would you describe your relationship with the healthcare team? What was the nurse’s role in your period in ICU? Are there any episodes/situations that have particularly impressed you when you think about your relationship with the nurse?

Questions relating to the experiences, perceptions and unexpressed needs of patients in ICU:

What were your feelings when you were admitted to the ICU? How did you experience those days in the ICU? Do you think the nurses met your needs? Do you feel they were able to understand you? Were they able to understand you even before you expressed your needs? During your stay in the ICU, did you feel cared for, supported, and reassured? Please, think about what an ideal nurse should be like. What kind of personal qualities and professional skills should they have?

### 2.7 Data analysis

In accordance with the qualitative nature of the study, no statistical techniques were used for data analysis. The transcripts of the interviews were analyzed according to the content analysis approach (Sandelowski and Barroso, 2002), which was carried out in several phases:

1. **Familiarization with the material:** Each transcript was read multiple times to gain an overall understanding and a deep insight into the participants’ testimonies.
2. **Independent analysis:** Two researchers (E.L., S.Q.) independently and simultaneously analyzed the transcripts by breaking down the text from general to specific, following the principle of horizontalization. Significant text units—words and/or phrases relevant to the research question—were identified.
3. **Labeling:** Each significant text unit was summarized using a descriptive label.
4. **Comparison and agreement:** The two researchers compared and discussed the labels. In cases of disagreement, a third researcher (N.V.U.) from the team provided the deciding opinion.
5. **Categorization:** Descriptive labels with similar meanings were grouped into categories. All labels across the interviews were aggregated by thematic relevance, allowing each category to be clearly defined and named.
6. **Refinement of categories:** Further comparison among researchers was conducted to distinguish the unique features of each category and to analyze differences between them.
7. Categories were then organized into broader thematic areas.
8. **Theory development:** A descriptive theory of the phenomenon was developed through final analysis of the categories and associated labels, aiming to capture and convey the meaning behind participants’ experiences.

**Figure 1:**
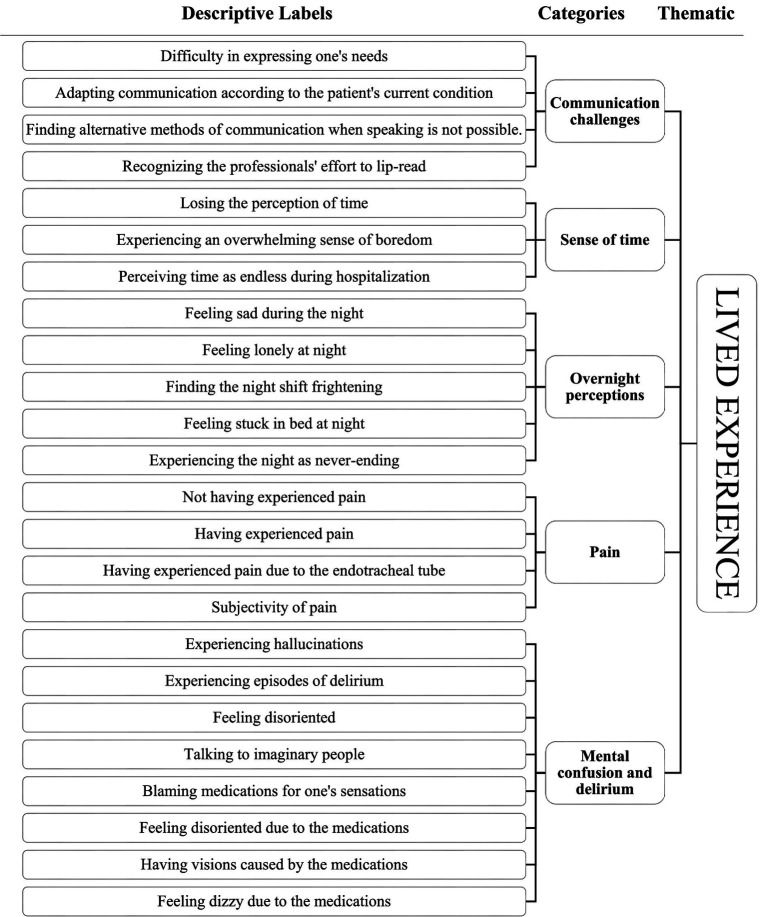
Example of analysis process.

In detail, an Excel spreadsheet was created to organize the categories alongside the significant text units that contributed to their development. This helped the researchers recall the detailed nuances of each text unit and, consequently, of the corresponding category. It also made it possible to include selected quotes from the participants in the study findings as excerpts. An example of the analysis process is provided in Figure 1. Audio recordings of the interviews and their verbatim transcription ensured accuracy and enhanced the credibility of the research material. Transcripts were not returned to participants for feedback. The transcripts were analyzed independently by two researchers (E.L., S.Q.) without the use of data analysis software. A constant comparison method was applied, involving repeated reference to the original transcripts and significant text units to ensure that each level of analysis remained faithful to the participants’ words and the original material, thereby maintaining neutrality. In cases where consensus could not be reached, two additional researchers (N.V.U., C.M.) were consulted. Throughout the study, the researchers kept a research diary containing methodological and reflective notes. These diaries documented each step of the process, including critical moments, and were used to develop awareness of potential biases and preconceptions, thus helping to minimize their influence during data analysis. The research group also reviewed and analyzed these diaries. Consistency was ensured by considering the full range of experiences described by participants, including atypical or less common situations. Member checking of the final findings was not performed.

### 2.8 Ethical considerations

The study protocol, any amendments, the informed consent form, the consent for data processing, and all patient-related information were approved by the local Ethics Committee (CE-AVEC – Comitato Etico Area Vasta Emilia Centro) on 06/06/2022. To participate in the study, each patient was required to provide written informed consent, including consent for the processing of personal data. Informed consent—along with consent for audio recording and data processing—was obtained by the CTV ICU research team 1–2 days after transfer to the CSU, for all patients who met the inclusion criteria. This followed a detailed explanation of the study and its objectives.

### 2.9 Rigor and reflexivity

Methodological rigor and data reliability were ensured through multiple strategies. With prior consent, interviews were audio-recorded and transcribed verbatim into Word documents. Transcripts were pseudo-anonymized using codes assigned to participants at the time of enrollment. Once the accuracy of each transcription was verified, the corresponding audio recordings were deleted. The Word files containing transcripts, memos, field notes, and the researchers’ diaries were stored on a password-protected computer accessible only to the study team. Socio-demographic information was stored in separate files, distinct from the interview data. All study-related materials will be retained for 5 years, as stated in the informed consent form. This study has been reported in accordance with the Consolidated Criteria for Reporting Qualitative Research (COREQ) (Tong et al., 2007).

## 3 Results

### 3.1 Characteristics of participants

The study included a total of 21 participants who underwent heart or lung transplants, as detailed in Table 1. Among them, 18 participants (86%) had received heart transplants, while 3 participants (14%) had undergone lung transplants. The mean age of heart transplant recipients was 57 years (SD ± 8.5 years), with 78% (*n* = 14) male and 22% (*n* = 4) female. Lung transplant patients had a mean age of 44 years (SD ± 7.2 years), with 67% (*n* = 2) male and 33% (*n* = 1) female. The average length of stay in the intensive care unit was 7 days (SD ± 3.5 days) for all participants. Specifically, heart transplant recipients stayed in the ICU for an average of 6 days (SD ± 2.8), while lung transplant recipients had a longer stay, averaging 13 days (SD ± 4.1). Post-operative complications were reported in 4 participants (19%): 3 among heart transplant patients (17%) and 1 among lung transplant patients (33%).

**Table 1:** Characteristics of participants.

Characteristic	Total (*n* = 21)	Heart transplant (*n* = 18)	Lung transplant (*n* = 3)
Mean age (± SD)	54.2 (± 9.3)	57 (± 8.5)	44 (± 7.2)
Gender (male)	16 (76%)	14 (78%)	2 (67%)
Gender (female)	5 (24%)	4 (22%)	1 (33%)
Mean ICU stay (± SD)	7 Days (± 3.5)	6 Days (± 2.8)	13 Days (± 4.1)
Post-operative complications	4 (19%)	3 (17%)	1 (33%)

### 3.2 Thematic findings

The thematic categories were developed through a detailed analysis of patients’ narratives, which explored their experiences starting from the guiding questions posed by the interviewer. However, many themes often extended beyond these initial questions, as patients freely expressed and elaborated on topics that were most meaningful to them. This open and flexible approach allowed for a richer and more authentic understanding of their perspectives. The analysis yielded six main themes: care environment, sensorial perceptions, person’s empowerment, lived experience, transplant path, quality of care provided and 26 categories (Table 2).

**Table 2:** Themes and categories found.

Themes	Categories
Care environment	Sounds and noises
	Isolation
Sensorial perceptions	Communication challenges
	Sense of time
	Overnight perceptions
	Pain
	Mental confusion and delirium
Person’s empowerment	Motivational drives
	Relationship with loved ones
	Faith and religious beliefs
Lived experience	Memories
	Awakening
	Patient’s feelings and mood in ICU
	Weakness and frailty
	Thoughts on the donor
	Stay in ICU
Transplant path	Pre-transplant
	The transplant
	Post-transplant
Quality of care provided	Actions, thoughts and feelings of caring
	Relationship with staff
	Health care needs
	To be informed
	Patient’s perception of ideal nurse
	Patient’s view of staff and hospital
	Healthcare research

### 3.3 Care environment

The care environment profoundly impacted transplant patients, who vividly described multiple sensory and physical components they encountered during their ICU stay. Key elements of this environment included the visual and auditory stimuli, the physical layout of the hospital rooms, and the emotional effects these factors elicited. The care environment left a strong impression on transplant patients, who frequently described what they saw, heard, and perceived during their ICU stay. The interviews highlighted their experience of isolation, the various sounds and noises, and the physical characteristics of the hospital rooms, as well as the sensations these elements evoked. Patients were placed in individual isolation rooms to reduce the risk of infection and protect them from the external environment. However, this physical isolation also carried negative emotional implications. Several participants expressed feelings such as:

“I really felt alone” Interview n.14 (I.14).

“I felt a bit abandoned” I.13.

At the same time, many patients acknowledged the protective value of isolation. One participant shared:

“I felt protected by the environment where I was” I.8.

The recovery experience was also marked by the constant presence of sounds, especially monitor alarms, which some patients described as an inescapable part of their daily experience:

“Sounds, always lots of sounds. I heard sounds. I heard many sounds, I was the only one who could hear them” I.1.

### 3.4 Sensorial perceptions

Participants reported that their treatment was marked by intense sensory experiences, including pain, delirium, altered perceptions of time, and communication difficulties. The experience of pain varied significantly among individuals. Some reported no pain, while others experienced discomfort related to the endotracheal tube or prolonged immobility. One participant shared:

“I was immediately amazed, I didn't feel any pain” I.4.

Several participants described a sense of disorientation and confusion, feeling almost alienated from the outside world. Factors such as an altered sense of day and night, the isolation required for protective purposes, sleep deprivation, and continuous drug infusions contributed to sensory disturbances like hallucinations, delirium, and disorientation. For example:

Some patients said:

“I realized that I was talking to a lot of people: to my imaginary friend or my dead mother” I.13.

“I think that is, you say, uncontrolled delirium, it's something you can't control” I.13.

“I felt inside a bubble. Inside a completely opaque bubble, it was a strange sensation” I.5.

The interviews also emphasized how patients experienced time as distorted—dilated and seemingly endless—until they lost their sense of its passage. Nighttime, in particular, was described as a frightening and emotionally intense period. For many, the night became a moment of deep reflection, where the absence of loved ones was felt most strongly. Participants described it as follows:

“I was very happy, but when night came—we're talking about just six nights—they felt very long” I.14.

“Sometimes I felt even sadder, especially when I knew evening was approaching… the nights were very long, and after that, there seemed to be fewer staff around—you felt more alone” I.5.

These altered perceptions often resulted in unmet healthcare needs, many of which were not communicated to staff. Communication challenges were influenced by factors such as stress, sedation, intubation, disorientation, and isolation. As one participant explained:

“At first, I couldn’t speak, so I wrote things down to make myself understood. Some staff members could even read my lips or made an effort to do so…” I.1.

### 3.5 Person’s empowerment

Several times, patients emphasized the need to find motivational drives that could help them overcome anxiety and fear experienced during their stay in ICU. These motivational sources were both internal—stemming from the patients’ own attitudes—and external, often represented by family support and spirituality. In terms of personal coping strategies, some patients found comfort and relief by establishing relationships with healthcare professionals. For example, one participant shared:

“The best thing was that there was a woman with me, a healthcare assistant, who let me cry—she allowed me to release everything” I.3.

Other patients sought distraction and strength by mentally distancing themselves from the hospital environment, focusing instead on familiar or positive thoughts. As one participant expressed:

“I wasn’t confused—my mind was still working. I tried to think about work, about other things… It’s just who I am: I never give up. I can always find some peace, a bit of serenity” (I.21).

Another participant added:

“If I don’t do it, no one else will do it for me. So, I’m here to get better” I.3.

Contact with family members was also described as a crucial source of motivation and emotional support:

“You need to be with your loved ones. Even if we couldn’t touch each other, you could still feel their presence. That helps you hold on” I.14.

Finally, several participants found hope and comfort through spirituality. One patient expressed this sentiment as:

“There’s someone up there who’s helping me” I.6.

### 3.6 Lived experience

The interviews enabled an in-depth analysis of patients’ memories, which were closely tied to a wide range of emotional experiences. While some patients recalled their ICU stay clearly, others struggled to remember events and had to reconstruct their experiences with the help of family members. As two participants explained:

“My family told me about the critical days—I remembered very little myself” I.1.

“I didn’t remember it, but they told me the surgery was short. It lasted… well, the same day, around six-thirty… maybe eight or nine… and it was already over, and everything went well” I.2.

The most confused and difficult memories to describe were often related to the awakening phase, which was commonly experienced as slow and disorienting, with a distorted sense of space and time:

“I was always asleep, I didn’t notice anything. On the second day, I started to wake up a little—I found myself in a strange place” I.4.

As patients revisited their experiences, both positive and negative emotions emerged—sometimes even simultaneously. Several participants described strong feelings of anxiety, fear, panic, and agitation:

“Fear was a feeling that stayed with me from beginning to end” I.3.

“Well… definitely negative emotions, like the anxiety of feeling at the mercy of events. I couldn’t control anything—I was lying in a bed, I couldn’t move, not even to get a glass of water. And even if I managed to, I couldn’t drink it properly because the water would choke me… so yes, I definitely felt a lot of anxiety, a lot of fear. The first two days, there was this irrational fear” I.14.

One participant found it difficult to define their emotions clearly, describing instead a state of inner confusion:

“My feelings weren’t clearly defined… I didn’t know if I was out of it, if I was strange, if I was lucky, or what… I was so shaken—can I say that? I couldn’t measure joy or pain, or the opposite” I.7.

Despite these negative emotions, some participants expressed positive feelings, such as happiness and a sense of gratitude or good fortune:

“I was and still am happy, even though I wasn't fully out yet” I.2.

“Yes… and it came true… I couldn't ask for more, I was here, I felt good, I was well cared for” I.21.

“And I realized—always with tears—the value of this opportunity. The rest would be what I deserved. I’ve always been lucky, and now even luckier” I.15.

Among the various reflections, many patients expressed hope for the future, particularly in relation to the outcome of the transplant and the prospect of improving health:

“The only thing I hoped for was that, with all the days ahead of me, my health would keep improving” I.20.

“On one hand, I hoped not to return to ICU” I.5.

In addition to reflecting on their own experiences, several patients also spoke about their thoughts and emotions concerning their donor. While some were curious about the donor’s identity, others expressed feelings of guilt, or simply imagined the person who had made their transplant possible:

“There was this strange feeling—knowing that I was still here while someone else wasn’t. I wasn’t sure if it was guilt or if it was okay to feel that way” I.13.

“I asked doctors and nurses: 'Can I know something about the heart?… I was just a bit curious” I.15.

Although many participants described their ICU experience as difficult and prolonged, positive reflections were also common. Some reported having few negative memories and recalled a high level of care and respect from staff:

“It was all positive… the care I received… everything was positive” I.6.

“I experienced everything positively. Everything was good. Like I said, maybe there was just that one rude response that shouldn’t have happened — it really depended on the person, the mood, the moment…” I.15.

“Nothing to complain about, only good things — I’m telling you the truth. I felt great, they respected me, treated me well, everything went smoothly” I.18.

### 3.7 Quality of care provided

The interviewees reported experiences related to nursing gestures and the actions taken to meet their needs. The needs identified by the patients were varied and involved both the sensations they experienced in the ICU and their direct requests to healthcare personnel. Some unpleasant feelings of discomfort were mentioned, mostly due to prolonged bed rest and immobility, along with sensations of cold and thirst:

“Well, the feeling was not the best. You had to stay still, you could barely move in bed… the constant oxygen, the constant dryness… these are things I will remember. And then the uncomfortable beds… maybe they sound like minor issues, until you actually go through them… you have to experience them to understand” I.5.

Most of the respondents stated that nursing staff responded to their needs promptly—whether it was the need to vent, to move, or to be hydrated—and sometimes even anticipated those needs.

Participants shared the following perspectives:

“They anticipated my needs a lot, they came to check if everything was okay if I had all the connections attached” I.18.

Gestures of care made patients feel welcomed, reassured and cared for—allowing them to feel seen as individuals and to benefit from the continuous presence of the healthcare team.

The attention and compassion of nurses were often expressed through small but meaningful actions:

“I needed to drink, or at least to quench my thirst, so I asked for a popsicle. They went and got me one. I really can't complain…” I.14.

“Okay, well… I’ll remember those popsicle days, believe me. Call me pathetic, call me whatever you want. But those are the things that stay with you” I.7.

On the other hand, in two interviews, the need to feel more understood and listened to emerged—particularly in patients with a longer and more complex medical history:

“Sometimes they were more present, sometimes a little more distant. Sometimes very present, sometimes less so… but overall, they were there” I.20.

“I would have liked more availability, more understanding” I.8.

The interviewees also clearly expressed their desire and need to be informed about the various stages of the transplant process, in order to feel reassured:

“To feel calm, I need to know exactly what I’m facing, step by step…” I.14.

On the other hand, only one interviewee said he/she preferred *not* to be informed about everything, believing that staying “in the dark” helped him/her face hospitalization more calmly:

“It was better not to know everything in detail. For example, at one point they had to remove the drains… which was really tough. I didn’t know that was coming, and so I stayed calm” I.11.

In addition to discussing gestures of care and the needs related to being cared for, the interviews also explored the characteristics of the ideal nurse and the role nurses play in patients’ lives. Most respondents described positive experiences with the nursing staff, highlighting empathetic relationships built on trust and respect. Some even said the nurses felt like family, appreciating their constant presence and readiness to help.

One interviewee expressed this sentiment as follows:

“We weren’t so formal with the health personnel; They called me by name, joked around and always asked me ‘how are you?’. All the time” I.3.

When asked, *“Please, think about what an ideal nurse should be like. What kind of personal qualities and professional skills should they have?,”* most interviewees emphasized a balance between technical competence and human compassion. The nurse was seen as someone who stands beside the patient, who puts themselves aside in order to care for others. For some, nursing was described as more than a job—a true vocation:

“First of all, a nurse must love… I mean, they must really enjoy what they do. It’s not about the salary—it’s almost a mission. I think the perfect nurse is someone who sees a bit of themselves in the patient” I.13.

Participants highlighted the individuality of each professional—recognizing that this diversity made them special, complementary, and effective as a team. At the same time, the nurses’ competence, dedication, and attention to care were widely praised:

“Each of them really had something special. You can’t all be perfect, but together they balanced each other out” I.1.

However, not all feedback was entirely positive. A few interviewees noted that some healthcare workers showed little empathy, simply performing their duties without emotional involvement:

“There was also a negative side. Some didn’t care—they just did their job, and that was it. On the positive side, if you asked something, others would take the time to answer and make you feel understood…” I.2.

The interviews focused not only on the characteristics of the health personnel, but also on the quality of the health facility and the services provided.

Overall, the quality of care services and the skills of the healthcare professionals were appreciated by the majority of patients. Most felt confident that they were receiving the right treatment in the right place:

“They did the best they could for me” I.15.

Additionally, though mentioned only in one interview, the importance of ongoing research and training in healthcare was acknowledged and valued:

“In my opinion, it's important to take time to reflect and contribute to the research process. Research is very important to me, and S. Orsola is a cutting-edge hospital” I.12.

Therefore, through the analysis, several unmet needs related to nursing care from the patients’ perspective have been identified. While physical needs such as mobility, hydration, and comfort were generally well addressed, emotional and informational needs were less consistently met. Some patients expressed a desire for greater empathy, understanding, and more consistent emotional support from nursing staff, particularly those with complex or prolonged hospitalizations. Additionally, the need for clearer, more comprehensive communication about the transplant process emerged as essential for reducing patient anxiety and fostering reassurance. Finally, a few patients noted a lack of personal connection with some nurses, highlighting the importance of compassionate engagement beyond routine clinical tasks.

### 3.8 Transplant path

The transplantation experience, as a whole, carries different meanings for the study participants. Several interviewees agreed on the traumatic and sudden nature of this experience, as illustrated below:

“The experience was dramatic. I had been waiting four years for a heart, and I was in top form, overjoyed. I was writing a book, doing sports, baking bread… the heart surgeon called me and told me there was a heart for me… I was suddenly plunged into this new reality, which was a bit shocking” I.11.

One patient, on the other hand, emphasized its strongly positive nature:

“I would not call my experience good, but let's say, yes, very good” I.6.

Transplantation is often experienced as a sort of revenge, highlighting the exceptional nature of the event:

“The fact of having a new heart should be seen as revenge and a new life, because I was no longer living, attached to a machine, so I saw it as a new life opportunity—new and real. Not that it wasn’t before, but it was at 30 per cent. Now instead, with the new heart, I see myself with a new life at 100 per cent. So when I come out, I will be a new me” I.3.

“I have a new heart! I have a new heart! How could I not be happy?” I.3.

In addition, one participant described the transplant as a gift from above:

“It was a real blow from heaven” I.20.

Interviewees shared different perspectives on their experiences both before and after the transplant. While waiting for the procedure, most patients felt the burden of their illness and the decline of their health, resulting in a sense of disadvantage and poor quality of life:

“All I know is that I got almost to the end and I felt really bad, I couldn't take it anymore, it was all a combination: my heart, my kidneys, my thyroid… then luckily they called me from Bologna and there was a heart” I.16.

“I was in bad shape… there was no telling, let's be honest, how long I had left to live” I.3.

For these reasons, several participants reported experiencing the wait for the transplant with excitement, especially once they received news of the availability of a suitable organ:

“When the surgeon came in the afternoon to tell me there was a heart for me, and then I was operated on the next morning… I never slept the whole night…” I.3.

“And so I couldn't wait. Just when they were about to take me to the operating theater, I felt like I was reborn” I.2.

At the same time, various meanings were attributed to the experiences that occur in the immediate post-transplant period. Participants described themselves as immediately enthusiastic about the opportunity they had received and reported that they perceived a change in their lives compared to pre-transplantation period. Patients said they immediately attributed another meaning to their own life:

“Since the day after I got the transplant, my life has changed. For three years I couldn't cross my legs because they were always swollen. Since two days after I was in ICU, I have been able to cross them instead!” I.12.

“So, after the transplant I was basically off-limits for two days, I mean I was not awake. When I woke up…because they wake you up very naturally, they don't wake you up all at once. You understand that something has changed. For example, breathing, for instance. And you realize that you are starting to live differently” I.3.

For some, waking up from anesthesia felt like a dream, and they only became aware of what had happened a few days after the transplant:

“When I woke up from the anesthesia, I felt inside a matted bubble, it was a very strange feeling. I heard voices but I didn't understand where I was, I couldn't realize. The nurses told me: ‘You're in intensive care, you've been operated on, we've transplanted you’… but I didn't think about what had happened… I just didn't understand. I understood it only after days. I'm really understanding it now after days” I.5.

One participant uniquely reported being able to perceive the rhythm of his own heartbeats again:

“One of the things I remember most vividly is the physical sensation of going at 115 beats per minute” I.11.

Another respondent, meanwhile, reported a feeling of ‘ownership’ of the new heart:

“I feel sorry for those who lost a loved one to give me life, but now the heart is mine and I am not giving it to anyone. You become selfish in these situations” I.16.

On the other hand, many interviewees reported knowing, despite the importance of what had happened to them, that their journey would still be long and that the transplant was not an endpoint but a new beginning.

“When I get out of the hospital, I will know that it is not over, because, in any case, there are pills I will have to take for life, visits every few months…” I.3.

Often the idea of having overcome the disease and having taken revenge resurfaced.

## 4 Discussion

This study provided a profound insight into the multifaceted experiences of patients undergoing heart and lung transplantation, allowing to explore not only their psychological and emotional states but also the perceived quality of care during the ICU stay and immediately after. Our findings extend current literature by highlighting the effectiveness of conducting interviews shortly after ICU discharge, a timing that contrasts with some previous works. Patients’ narratives revealed the immediate aftermath of their intensive care experience while memories were still fresh, yet often fragmented and disorganized. For instance, many patients vividly described their sensory perceptions, such as pain and delirium, as well as difficulties in perceiving time accurately—a common theme supported by Del Barrio et al. (2004), who attributed cognitive alterations to the disrupted sleep–wake cycle. One patient shared: “*I felt inside a bubble. Inside a completely opaque bubble, it was a strange sensation*,” illustrating the temporal disorientation that pervades the ICU experience. This aligns with our findings that time perception was frequently distorted, impacting patients’ cognitive functioning but leaving their core thoughts and emotions largely intact. Communication barriers were frequently cited as significant obstacles. Several participants mentioned moments when they struggled to express themselves, leading to feelings of frustration and isolation. These accounts support Freeman-Sanderson et al. (2025), who argue that ineffective communication in the ICU may negatively influence patients’ recovery. Not all interactions were positive; some patients perceived care as “cold” or “purely technical,” pointing to the need for consistent empathetic communication to avoid further distress. In this instance, a patient stated: “*Some did not care—they just did their job, and that was it.”* The preservation and reconstruction of memories emerged as a vital element in patients’ psychological processing of their ICU stay. Echoing Maartmann-Moe et al. (2021), many patients reported either clear recollections or “confused memories from the awakening phase.” One interviewee explained, “*I was always asleep, I did not notice anything. On the second day, I started to wake up a little*.” This suggests that the interviews themselves served a therapeutic purpose, enabling patients to organize fragmented memories into a coherent narrative and thus begin making sense of their experiences. Isolation during ICU hospitalization was another complex theme, experienced ambivalently by patients. Many expressed feelings of protection due to isolation (“*I felt protected by the environment where I was”*) (Biagioli et al., 2017), but also a deep sense of loneliness and abandonment, mirroring Lee and Halliday’s (2025) descriptions of ICU environments as claustrophobic and frightening. One patient said: “*I felt a little abandoned*.” The literature, including Saliba et al. (2022), emphasizes patients’ lack of preparation for the psychological challenges of isolation, a gap our findings also highlighted. Patients expressed a strong need for more information to reduce anxiety related to isolation, yet interestingly, one patient noted: “*It was better not to know everything in detail. For example, at one point they had to remove the drains. which was really tough. I did not know that was coming, and so I stayed calm*.” This underlines the necessity for tailored communication strategies that respect individual preferences. The transplant experience was universally perceived as a profound life transformation. Nour El Hadi et al. (2025) described this process as “recovering life,” a sentiment echoed by our participants who viewed the transplant as “*a new life opportunity*” or “*a real blow from heaven.”* At the same time, the emotional and symbolic meaning of the donor organ emerged strongly. Feelings ranged from profound gratitude to guilt (“*I feel sorry for those who lost a loved one to give me life”*) and curiosity, revealing a complex psychological dimension often overlooked in clinical care. Patients’ reflections on the donor highlight an area for future research and support interventions. All these feelings, reflect the emotional complexity of post-transplant adaptation. Patients reported increased appreciation for basic functions such as walking and breathing, feelings that intensified upon ICU discharge with emotions of “liberation,” “joy,” and sometimes “euphoria,” confirming Nour El Hadi et al.’s (2025) findings on the rebirth-like nature of the transplant event. Emotional responses during the interviews were intense and revealing. Trembling voices and tears highlighted the depth of feelings experienced—ranging from fear of surgery and uncertainty about the future, to gratitude and happiness. One patient described the transplant journey as: “*My feelings were not clearly defined… I did not know if I was out of it, if I was strange, if I was lucky, or what… I was so shaken—can I say that? I could not measure joy or pain, or the opposite*.” This raw emotional expression emphasizes the importance of healthcare providers recognizing and addressing psychological needs throughout the transplant process. The role of healthcare professionals, especially nurses, was central to patients’ experiences. Participants valued not only technical competence but, crucially, relational skills. One patient reflected, “*Okay, well. I’ll remember those popsicle days, believe me. Call me pathetic, call me whatever you want. But those are the things that stay with you*.” Such testimonial aligns with Thain and Gibbon (1996), who emphasize the positive impact of empathetic relationships in ICU settings. Participants reported being assisted in a setting that generated gratitude, along with positive emotions and courage to face the situation. Most of them received a prompt response to their care needs by creating an empathetic and almost confidential relationship (*They called me by name, joked around and always asked me “how are you?.” All the time*). Only a few reported occasional inconveniences that occurred with healthcare personnel, complaining about assistance that was sometimes distant and inattentive. Overall, they believe that the ideal nurse is the one who provided them with care, possessing both technical and relational skills and the ability to demonstrate closeness. From the words of the patients, it emerges that relational skills make the difference and are fundamental in the care path. One patient stated: “*Each of them really had something special. You cannot all be perfect, but together they balanced each other out*.” The need to have someone by their side is what is highlighted by the interviews, whether it is a healthcare professional or a caregiver. Family presence was identified as essential for emotional support and motivation during hospitalization and recovery. Consistent with Thain and Gibbon (1996), participants viewed relatives as vital anchors helping to mitigate the isolating effects of ICU admission. One patient said, “*You need to be with your loved ones. Even if we could not touch each other, you could still feel their presence. That helps you hold on*.” This underscores the importance of policies facilitating family involvement to promote psychological well-being. In conclusion, this study reinforces the necessity of integrated care approaches that address the clinical, emotional, and relational dimensions of transplantation. The vivid testimonies reveal the importance of empathetic communication, family involvement, and psychological support tailored to individual patient needs to improve recovery and quality of life post-transplant.

### 4.1 Strengths and limitations of the work

This study has some limitations that should be considered. Firstly, the study was conducted at a single university hospital in Italy, specialized in cardiac and pulmonary transplantation. For these reasons, the findings may be applicable only in similar highly specialized settings. Additionally, the interviews were carried out within a few days of the participants’ discharge from the ICU. While this timing allowed the collection of fresh memories, it may have limited the participants’ ability to fully process and articulate their experiences. Some participants reported fragmented or incomplete recollections of their ICU stay, likely influenced by the effects of sedation, delirium and the acute nature of their condition. This may have impacted the depth of the data and introduced variability in the findings. Furthermore, the diverse backgrounds and experiences of the participants, particularly regarding their emotional and psychological responses to the transplant process, may have influenced how they perceived and reported their needs and challenges during hospitalization. Another limitation lies in the fact that there was no selection or stratification of participants by age. This may have affected the findings, as age can significantly influence both physical and emotional responses to intensive care experiences, as well as the ability to articulate these experiences. The study design included a single interview per participant, and the participants did not have the opportunity to review or validate their transcripts. This could limit the accuracy and completeness of the narratives. However, efforts were made to ensure the rigor and credibility of the findings through strategies such as detailed data analysis and adherence to data saturation principles to confirm representativeness.

### 4.2 Recommendations for future research

Based on the findings from the literature and the data collected through patient interviews, it is evident that raising awareness among healthcare professionals on this topic is essential for delivering personalized, high-quality care. In light of the significant psychological impact associated with the transplant experience, as consistently highlighted in the literature, it would be beneficial to implement a targeted training program focused on the psychosocial dimensions of patient care. Such a program should be offered to the entire multidisciplinary healthcare team—not only nurses, but also physicians, physiotherapists, and healthcare support workers. Moreover, valuable insights for improving clinical practice could be gained through an additional qualitative study involving interviews with both patients and healthcare professionals, particularly nurses, on the same topics addressed in the current research. This approach would allow for a comparison between patients’ lived experiences and professionals’ perspectives, potentially revealing discrepancies or commonalities in perception. Such a comparison could foster a more comprehensive understanding of the care experience and support the development of practice changes that are more aligned with patients’ needs and expectations.

### 4.3 Implications for policy and practice

The findings of this research emphasize the importance of addressing the psychological aspect of transplant patients. This insight has direct implications for healthcare practice, particularly in improving patient care and staff education. Implementing a comprehensive training program for healthcare professionals, including nurses, doctors, physiotherapists, and social workers, is crucial for enhancing the quality of care and ensuring a more personalized approach to patient management. To incorporate psychological support for transplant patients into healthcare, policies should be updated to include regular workshops and courses focused on these needs as part of mandatory professional development. This would improve patient outcomes and promote more compassionate, comprehensive care. Healthcare institutions should also implement protocols that make psychological care a core element of post-transplant care.

### 4.4 Relevance to clinical practice

Building on these premises, this study aims to propose an innovative model of care for transplant patients, centered on the person and their perceived priorities. By integrating the patient’s perspective into organizational decision-making, this approach seeks to reshape care delivery in a way that is both patient-focused and aligned with institutional strategies. The study suggests that a shift in the care approach could redefine the hospitalization process across various levels of care intensity. This change could lead to more personalized, compassionate care, improving effectiveness, efficiency, and appropriateness, with significant human and economic benefits. By recognizing the experiences of transplant patients in ICU, healthcare providers can implement specialized staff training, introduce patient-centered interventions, and offer high-quality care that enhances patient satisfaction.

## 5 Conclusion

The present study allowed for the exploration of a wide range of perceptions, emotional experiences, and subjective meanings expressed by patients admitted to ICU following heart and lung transplantation. For the majority of participants, transplantation was perceived as a second chance—a renewed opportunity and the beginning of a new life. A prominent theme that emerged from this study is gratitude. Many patients conveyed a profound and enduring sense of thankfulness toward both the healthcare professionals, for their dedication and expertise, and the donor, for the extraordinary gift of life. Despite this prevailing sense of gratitude, the transplant experience was also marked by emotionally challenging moments, including feelings of loneliness, isolation, and a perceived disconnection from family members. These difficulties, however, were often mitigated by the attentive presence and emotional support of healthcare staff, as well as by patients’ ability to reframe isolation as a necessary and ultimately beneficial part of the recovery process. Consistent with existing literature, transplantation was described as a complex and transformative clinical pathway, in which the role of healthcare professionals is pivotal. Their ability to assume full responsibility for patient care, to provide both technical and emotional support, and to establish meaningful therapeutic relationships can significantly impact patients’ experiences and outcomes. Accordingly, the findings underscore the importance of promoting a humanized and patient-centered model of care, characterized by individualized attention to patients’ clinical and emotional needs. Such care should not be overlooked but rather actively recognized and valued as a core component of high-quality transplant care.

## Data Availability

The datasets presented in this study can be found in online repositories. The names of the repository/repositories and accession number(s) can be found at: https://doi.org/10.5281/zenodo.15639370. This set of raw data cannot be made publicly available as including sensitive information, but might be available to interested researchers upon reasonable requests to researchers.
